# Education and inspirational intuition - Drivers of innovation

**DOI:** 10.1016/j.heliyon.2021.e07923

**Published:** 2021-09-02

**Authors:** Jochem Wilfried Müller

**Affiliations:** University of Applied Sciences Ansbach, Faculty of Economics, 91522 Ansbach, Germany

**Keywords:** Education, Intuition, Creativity, Inspirational, Competences, Intelligences, Innovation, Perception, Assessment, Incubation, Illumination, Artificial intelligence

## Abstract

This paper deals with the fundamental relation and importance of education, intuition and creativity as innovation drivers. In this context, the central questions arise what is meant by “education” and what creative roles do “education” and “intuition” play in the emergence of the new?

The modern concept of education developed essentially on the ideas of humanism and enlightenment, which found their roots in antiquity. In the current understanding, education becomes a continuous adaptation and development. The human being is a dynamic entity, which is both influenced by the respective environment, and at the same time can itself change and influence this environment. Education generates and imparts knowledge throughout life. For perceiving and judging our environment and life, there is besides rationality also a world of feelings, emotions and heart.

Based on the perception and judgment model of Carl Gustav Jung, intuition and especially intuitive competence are addressed as central influencing factors of education and creativity. Intuitive perception opens us up to new knowledge and opens up a multi-level spectrum of creativity. As the driver of innovation, education becomes the link between conscious rationality and unconscious emotionality. A rational problem analysis should be followed by an intuitive solution finding, an instinctive and emotional grasp of future market opportunities. In this context, the inspirational intuition plays a particularly important role.

At the end of the paper there is a critical appreciation of the innovative possibilities of mankind. It is at the same time an exhortation for a preserving and integrative creative work. Our creative power gives us creative potential and enables us to change and shape the world over and over again.

## 1 Introduction

The great importance of education and inspirational intuition as an important holistic instrument of perception, assessment and innovative acting, has not yet been sufficient scientifically investigated. However, intuitive competence plays a prominent role in decision-making when data is scarce or ambiguous, in the grasping of networked and interdisciplinary relationships, and in orientation and solution development in complex situations (Müller and Rippel, 2011, p. 119). With the help of intuitive pattern recognition, we can quickly grasp and assess complex structures rationally and emotionally. Furthermore, intuitive competence helps us in creative thought processes, planning and forecasting in highly dynamic, networked or disruptive environments and as an aspect of intuitive communication in consulting, team and leadership relationships.

With the help of intuition, inventors, for example, can anticipate new business fields. Such forward-looking actions cannot usually be planned purely rationally, but require an “intuitive feel”. Visions and imaginations open up options for action beyond the traditional und usual solution paths. Mere rational thinking here would probably remain in old traditional patterns of action. The door for evolutionary or revolutionary ideas would remain closed without powerful education and inspirational intuition.

The main aim of this paper is to show the importance of education and inspirational intuition as drivers of innovation. While education should make existing knowledge available and convey it, inspirational intuition ensures the creation of non-existing, new knowledge. Only through the interaction of knowledge preservation and knowledge generation can a constant change and innovation be achieved.

## 2 Material and methods

This article is based on a selective literature research and intends a systematically compile and develop of scientific findings. In this context various publications on the topic of education and intuition have been consulted and evaluated. The systematic review focuses on historical development, definitional approaches and terminological delimitations. With the help of the content analysis, central results pointed out.

Complementing selective literature research, the article draws on Carl Gustav Jung's model of perception and judgment. For the interplay of rationality and emotionality, the psychoanalyst Carl Gustav Jung developed a model, that explains, how people relate to reality in different ways (Jung, 2008, p. 23 ff.).

Müller and Rippel went on to develop the CREA LEADERSHIP® intelligence model at Ansbach University of Applied Sciences, relying in particular on intuitive competence as a unique selling point (Müller and Rippel, 2011, p. 91).

As an additional methodological approach, the stage pyramid of creativity shows the different levels of creativity and in particular addresses inspirational intuition as a central success factor. Based on individual and collective creativity potential, creativity techniques can be applied. However, truly holistic thinking structures can only be used through intuitive approaches (Müller and Rippel, 2011, p. 281).

As a further method, the systematic theory of creative thinking according to Wallas is applied. The paper emphasizes the important bridging function of inspirational intuition between rational problem analysis and intuitive, emotional solution generation as the basis for prototyping and implementation (Wallas, 2015, p. 37).

## 3 Results

### 3.1 Intellectual historical development

The Latin term “humanitas” helps to interpret the term “education”. It describes “education” with humanity and philanthropy as the basis for thinking and acting. The modern concept of education essentially developed from the ideas of humanism and the enlightenment, which found their roots in antiquity. “Humanism”, as an ideal of society and education, is characterized by respect for human dignity. Very important in this context is also the chance to further education and development. This requires non-violence as well as the right and the opportunity to express one's own opinion freely. The central question was therefore, how can the individual live a life of freedom, recognition, equality and justice in a self-determined and rational way? Important representatives of humanism included Nikolaus von Kues, Erasmus von Rotterdam, Johann Gottfried Herder and Friedrich Schiller.

**Figure 1:**
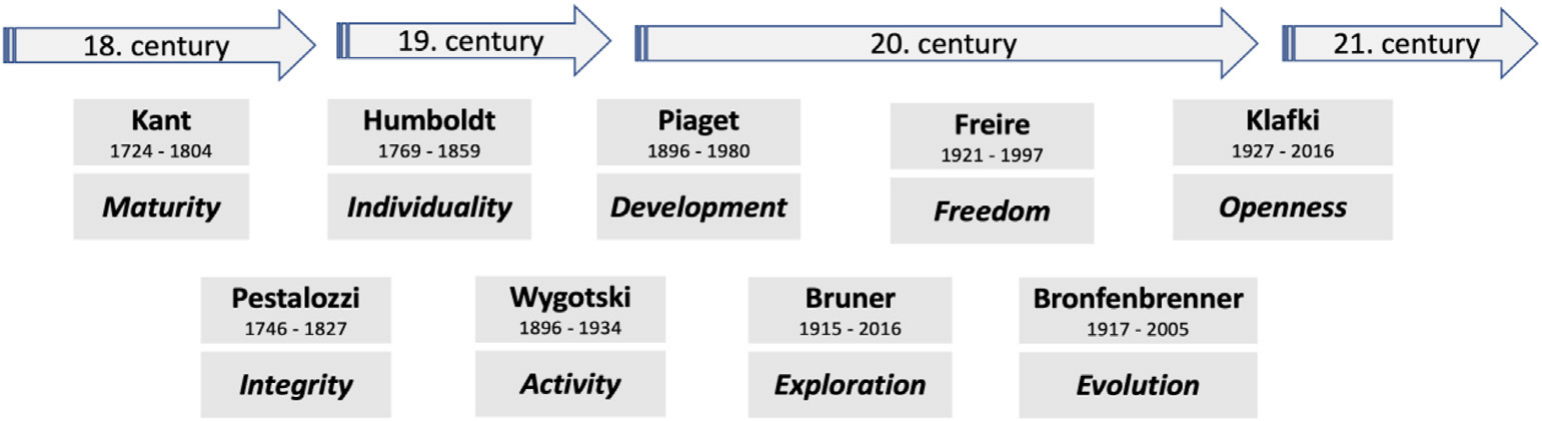
Core contents of education theory. Source: Own illustration.

At the end of the 18th century, or rather at the beginning of the 19th century, there was a development from classical education - as an education for usefulness - to the educational concept of the neo-humanists. They followed the idea that education emanates from the human being himself and must be determined less from outside. Wilhelm von Humboldt in particular had a significant influence on neo-humanist education. In his work of 1792, „Ideen zu einem Versuch, die Gränzen der Wirksamkeit des Staats zu bestimmen“, Humboldt also described the foundations and goals of his educational theory. “The true purpose of man [...] is the highest and most proportional formation of his strength into a whole”, postulated Humboldt (Humboldt von, 1851, p. 9; translated by the author). The perspective changes. It is no longer an educator who sets the goals determined by society, but the individual should form himself freely and self-determined (see Figure 1). The epoch of the enlightenment, as a pan-European intellectual movement of the 18th century, continued the ideas of humanism and at the same time set new emphases in terms of content. It was about man being “reasonable” and being able to solve his problems better according to the rules of reason. Thus, Immanuel Kant formulated the question in the Berlinische Monatsschrift of 1784, “Was ist Aufklärung”, and performed in the first two movements: "Enlightenment is man's exit from his self-inflicted immaturity. Immaturity is the inability to make use of his intellect without the guidance of another.” (see Figure 1) (Kant, 1784, p. 481; translated by the author).

Beyond the focus on humanity and rational maturity, the desire to view education from a holistic perspective grew. The name of Johann Heinrich Pestalozzi is particularly noteworthy here. His pedagogical approach, of “Kopf, Herz und Hand”, created a connection between rationality, emotionality and responsible action (see Figure 1). In his work „Nachforschungen“, of 1797, Pestalozzi formulated the interplay of investments, society and self-motivation as follows: “So I am a work of nature. A work of my gender. And a work of myself.” (Pestalozzi, 1797, p. 171; translated by the author).

From an international research perspective, the works of Lev Semyonovich Vygotsky, Jean Piaget, Jerome Bruner and Paulo Freire, in particular, led to an expansion of the understanding of education (see Figure 1). Vygotsky is considered the founder of the cultural-historical school and activity theory. As activity theory, it is a concept on object-oriented and object-mediated activity. Vygotsky found importance especially in American early education, with his concept of “zone of next development”. Vygotsky defines: “The area of not yet mature, but maturing processes is the zone of the child's next development” (Wygotski, 1987, p. 83, translated by the author). His research supported today's calls for creative early development of children as reservoirs of openness and resourcefulness. Jean Piaget's theory of cognitive development also supports the calls for early development of children's skills. Cognitive skills gradually emerge through sensorimotor development. We undergo a maturation process fueled by contact with the environment, social transmissions, and the pursuit of knowledge (Piaget, 1992, p. 8–11). Adapting to the environment and shaping the environment become central tendencies in becoming and being human. In particular, shaping the environment provides a link to inspirational intuition, as our original access point for systemic knowledge. The important role of inspirational intuition as a driving force of education, already emerged in Jerome Bruner's concepts of discovery learning or problem-solving learning and resulted in his didactic principle of the spiral curriculum (Bruner, 1970, p. 44). The curriculum arranges the learning material accordingly in the form of a spiral, so that individual topics are presented several times in the course of school education at a higher educational level and in different teaching forms. Consequently, the demands for lifelong knowledge acquisition and multimedia learning formats developed from this. In addition, the “little discoverers” in education should be encouraged in their thirst for knowledge at an early stage and their entrepreneurial thinking and spirit of initiative should be promoted. Paulo Freire understood education as granted freedom to discover, perceive and judge the world for oneself. Freire's thoughts can be understood as a harbinger of today's discussion about agility. He aimed at a dismantling of the hierarchical order between teachers and learners, as a form of learning equality. In this context, reality is not static, but should be understood as a process and characterized by constant transformation. He coined the concept of problem-formulating education and thus can already be understood as a mastermind of today's “creative problem solving.” (Freire, 2000, p. 32).

In the second half of the 20th century, educationalists further concretized the concept of education and continued the tradition of intellectual history. Important authors included Wolfgang Klafki, Hartmut von Hentig, Saul B. Robinson and Erich E. Geiszler. According to Wolfgang Klafki, the didactic goal is to provide people with an education that enables them to think and act critically, competently, self-confidently and in solidarity. Klafki speaks of a “categorical education”. In this learning process, the individual develops basic forms and contents of knowledge and understanding (see Figure 1). Man should interpret himself and his relationship to himself and the world and thus develop a well-founded action (Jank and Meyer, 2005, p. 216). The developmental psychologist Urie Bronfenbrenner describes the individual as a growing dynamic entity that is influenced by its environment and at the same time can itself change and influence this environment (Bronfenbrenner, 1981, p. 38). In this understanding education becomes a continuous adaptation and evolution (see Figure 1).

### 3.2 Definitional approach

There is no general and clear definition of education. Kerschensteiner, for example, defines: “Education is an individually organized sense of value awakened by cultural assets of individually possible breadth and depth.” (Kerschensteiner, 1926, p. 17; translated by the author). Kerschensteiner understands individuality as “the peculiar and unique way of action and reaction of the individual human being to the environment, as it is determined by heredity in its essence and has developed into a certain form through living conditions.” (Kerschensteiner, 1926, p. 4; translated by the author). This attempted explanation takes into account the scientific discussion, regarding the influence of genetics, environment and living conditions on the education of a person. The philosopher Henning Kössler defines: “Education is the acquisition of a system of morally desirable attitudes through the mediation and acquisition of knowledge in such a way that people define their location in the frame of reference of their historical-social world by choosing, evaluating and taking a stand, get a personality profile and gain orientation in life and action. Instead, one can also say that education creates identity.” (Kössler, 1989, p. 56; translated by the author).

Wolfgang Klafki, one of the best known, educational scientists of modern times, emphasizes another important task of education, “to enable the individual subjects to resist the demands and demands of society that are contrary to individual development. In this respect, critical faculties and role distance represent a central element of education.” (Klafki, 1991, p. 22; translated by the author)). Urie Bronfenbrenner developed a systemic and chronological perspective on human development. According to the central assumption of his model “Die Ökologie der menschlichen Entwicklung”, human development is understood as a process of progressive, mutual adaptation between the active, developing individual and the changing characteristics of his immediate environment (Bronfenbrenner, 1981, p. 37). “Development is therefore the “permanent change in the way the person perceives and deals with the environment.” (Klafki, 1991, p. 19; translated by the author). Bronfenbrenner's approach reflects a life-long process of development and change of the human being towards his systemic environment. Put simply, education consists of a lifelong process of acquiring multidimensional skills and abilities in order to use them for responsible action and design. Life-long education, in the phases of life fllowing childhood and youth, in further vocational training, adult education and personality development, is a continuous process of self-education of the whole person. Education generates and conveys knowledge throughout life. The human being therefore possesses the ability to form his own conscious judgement, to decide and to act. At the same time a sense of responsibility for the environment and the things he influences suffers from this (Mutzek, 2008, p. 50). Rational thinking is a prerequisite for analytical observation, scientific experimentation and logical reasoning. On this level of knowledge goals, strategies and tactics can be derived rationally. However, this purely rational thinking is only one side of human existence.

**Figure 2:**
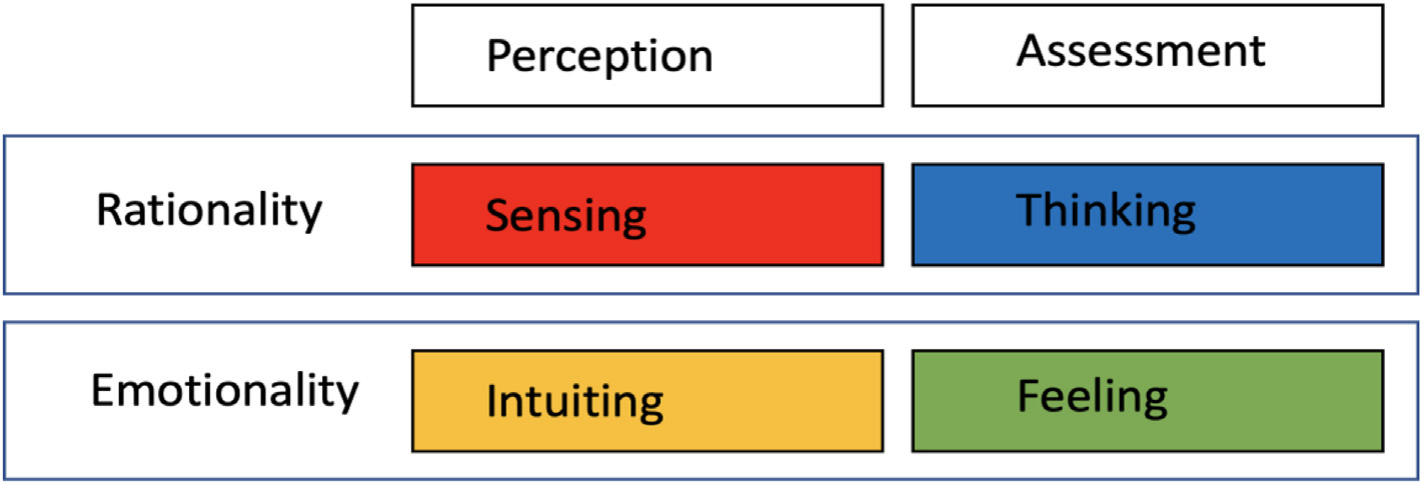
Four basic psychological functions according to Carl Gustav Jung. Source: Own illustration based on (Jung, 2008, S. 23 ff.).

For perceiving and judging our environment and life, there is still a world of feelings, emotions and the heart. For the interaction of rationality and emotionality, the psychoanalyst Carl Gustav Jung has developed a model, that explains, how people relate to reality in different ways (Jung, 2008, p. 23 ff.). The two dimensions “perception” and “assessment” play a central role in this process. Within the axis of perception Carl Gustav Jung distinguishes between “sensing” and “intuiting”, within the axis of judgment between “thinking” and “feeling” (see Figure 2). According to Jung, people perceive different impressions through their senses and use them to create their own world. Existing impressions are registered by sensory organs and an individual image is generated.

While Carl Gustav Jung was still strongly focused on the human sensory organs, today technical extensions of our sensory organs are particularly important. Technical sensors, detectors, transducers or probes, such as chemical analysis methods, microscopes, satellites, high-resolution cameras, thermal examinations, allow us to record and evaluate reality much more precisely, comprehensively and regularly. The foundations for education and thus for knowledge generation are expanding by leaps and bounds, opening up completely new analysis and forecasting methods for goal-oriented action. Sensory and technical perception is becoming a central survival and competitive factor. Jung sees “intuition” as another important source for humans to perceive their reality. He thus refers to the mental function with which we can discover the world of the possible, in the sense of a presentiment. The function of “foreboding” describes the ability to realize what is possible and what is not possible at a given moment. With intuitive perception we expand our field of perception. Intuition opens up a wider range of perception and thus supports our creativity. In the assessment dimension, we reflect on the reality perceived through sensation or intuition. According to Jung, two different techniques can be used for evaluation: mental ordering (thinking) and emotional evaluation (feeling). Intellectual ordering means forming categories and building grids into which the facts are placed. This process, which can be described as logical thinking, implies clear differentiation criteria and meaningful classification criteria according to content. In contrast, the emotional evaluation can be understood as an inner view. An emotional attitude or attitude towards what is perceived. “Vitarative” means, in reference to the Latin expression “in vita videre”, to “see life with the heart” and to judge the perceived sensory impressions emotionally. The most quoted sentence from the work “The Little Prince” by Antoine de Saint-Exupéry “You only see well with your heart. The essential is invisible to the eyes.” (Leitgeb and Leitgeb, 1950, chapter XXI.; translated by the author), expresses this inner perception metaphorically well. It complements the rational view and enables an emotional assessment of what is perceived.

**Figure 3:**
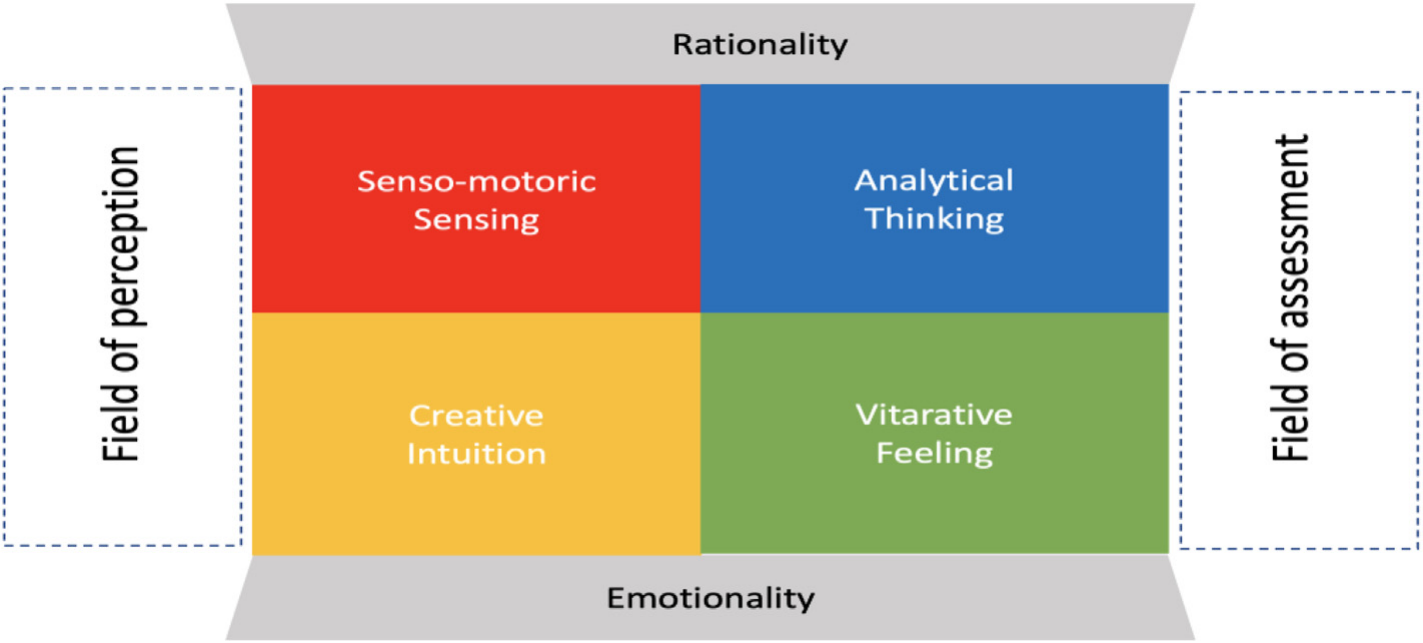
CREA LEADERSHIP® perception and assessment model according to Carl Gustav Jung. Source: Own illustration adapted from (Müller and Rippel, 2011, p. 64).

If one summarizes the considerations in the style of Carl Gustav Jung, one can see in simplified form how people deal with reality and derive options for action from it. Perception (feeling, intuiting) and assessment (thinking, feeling) form the foundation of his model. If the model is continued (see Figure 3), a field of perception between “senso-motoric sensing” and “creative intuition” and an evaluation field between “analytical thinking” and “vitarative feeling” can be identified (Müller and Rippel, 2011, p. 63–64).

### 3.3 Creativity, intelligence and inspirational intuition

Creativity helps to expand existing knowledge and constantly fuel the educational process. The Nobel Prize winner for physics, Prof. Dr. Gerd Binnig, describes creativity as “the ability to evolve” (Binnig, 1989, p. 20; translated by the author). In the creative process, new knowledge develops from existing knowledge in order to adapt to new circumstances and challenges. Lifelong education is thus understood as creative knowledge generation, in which old knowledge is continuously researched, acquired, reviewed and, if necessary, replaced by new knowledge.

**Figure 4:**
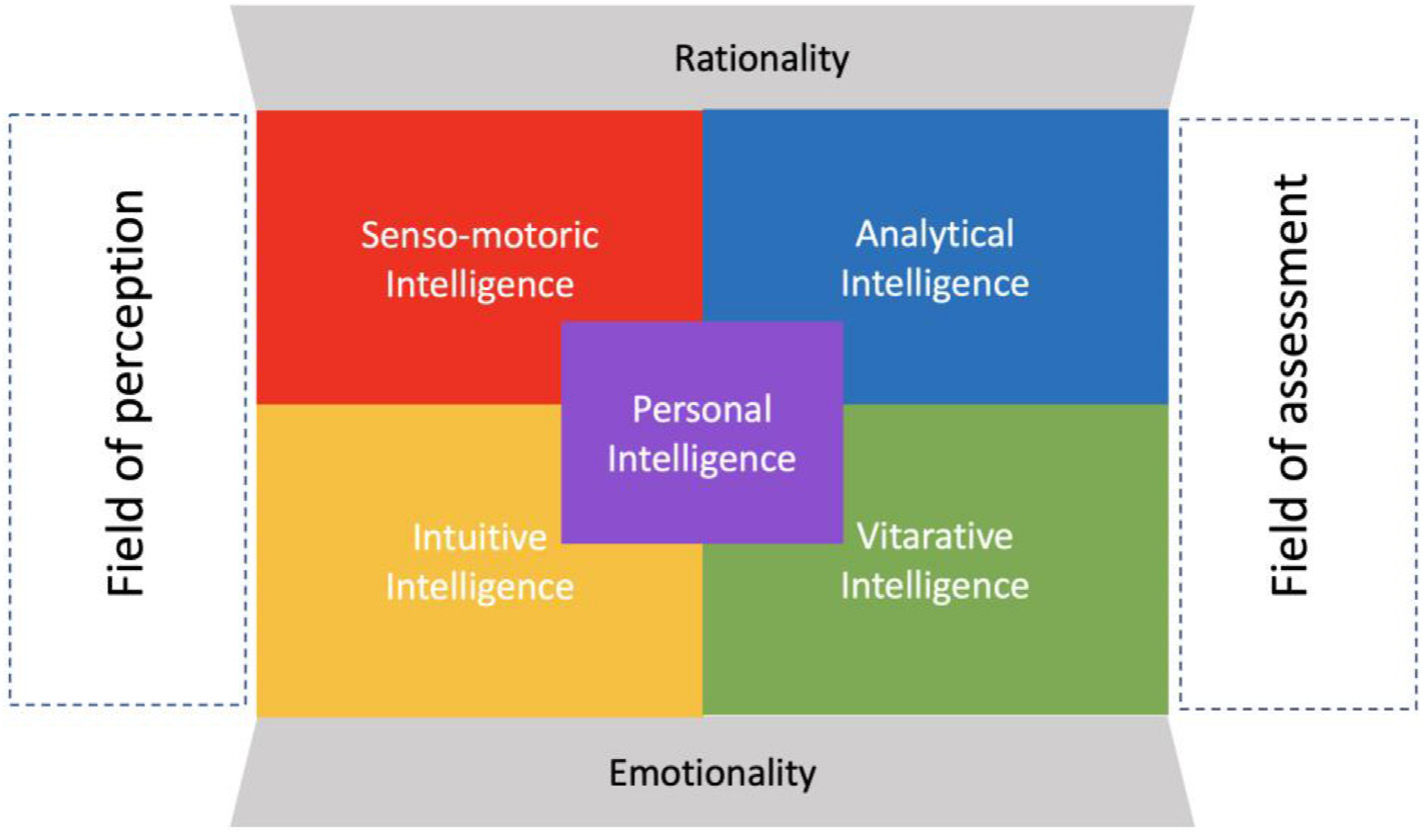
CREA LEADERSHIP® Intelligence model. Source: Own illustration adapted from (Müller and Rippel, 2011, p. 91).

Intelligence in its various forms plays an important key role in the acquisition of knowledge. In general, intelligence is a special form of perception, comprehension, understanding, judgment and action. The Latin origin of the word intelligence “intelligentia”, “intellectus”, “intellegere” means insight and cognitive faculty, mind and the mental ability to see, understand and implement something (Müller and Rippel, 2011, p. 79 f.). According to this, intelligence forms an important basis for the access and development of knowledge. The earlier and better one's own “knowledge stores” are filled, the better one can draw from them to generate new knowledge. For a long time, the classical intelligence quotient (IQ) was considered the dominant measure of intelligence. However, it turned out that the rational IQ intelligence measurement was too one-sided. Daniel Goleman recognized this and developed a second measurement unit for intelligence, emotional intelligence (EQ) (Goleman, 1996, p. 54 f.). He focused in particular on the dimensions of intra-personal intelligence and inter-personal intelligence. Intra-personal intelligence as a personal competence emphasizes self-perception and self-management as important factors for recognizing and handling one's own emotions. When dealing with other people, the most important thing is to be empathic, to empathize with others and to adapt emotionally to them. In addition, it is important to build and shape relationships and, if possible, to act together. Closely related to the concept of emotional intelligence is the term “social intelligence” originally coined by Edward Lee Thorndike. It focused on the ability to understand other people and to act wisely in interpersonal relationships (Thorndike, 1920, p. 227 ff.). In the early 1989s, Howard Gardner formulated his theory of "Multiple Intelligences” (MI). With his theory, Gardner breaks through the classical paradigm of one-sided intelligences and makes intelligence multidimensional. In the meantime, Howard Gardner differentiates more than a dozen different forms of intelligence.^1^ However, the complexity of the approach makes it difficult to understand intelligence and makes its practical application very complicated. Müller and Rippel, on the other hand, take up the considerations of Carl Gustav Jung and differentiate in their CREA LEADERSHIP intelligence model between rational and emotional intelligence. These, in turn, are further subdivided into perceptual and assessing intelligence. In detail, this results in the senso-motoric, the intuitive, the analytical and the vitarative Intelligence. In addition to these elementary forms of intelligence, there is a fifth, personal Intelligence (see Figure 4). Personal Intelligence helps us to realize ourselves through its superior, reflective and coordinating perspective. With it we can make decisions consciously and carry out actions or also consciously refrain from them, perceive and experience ourselves in them. Through self-reflection we open up the possibility to develop ourselves as a person and to live our strengths. Personal Intelligence also has the important task of defining prioritized goals in our lives, pursuing them or, if necessary, modifying or discarding them. We thus seek and follow our calling in life (Müller and Rippel, 2011, p. 90).

The individual forms of intelligence are not to be seen in isolation from each other, but rather are in an interactive relationship with each other and are mutually dependent. From birth onwards, the intelligence and knowledge stores begin to fill up. The experience and knowledge acquired in the course of life exert a direct influence on the intelligence and creative performance. If we ask ourselves the question which of the mentioned intelligences are particularly promoted in our educational system, it becomes apparent that the abilities of the left hemisphere of the brain are predominantly emphasized; while the abilities of the right hemisphere of the brain, especially imagination, intuition and creativity, fall behind. Especially intuition competence helps us in creative thought processes, planning and forecasting in highly dynamic, networked or disruptive environments and as an aspect of intuitive communication in consulting, team and leadership relationships.

**Figure 5:**
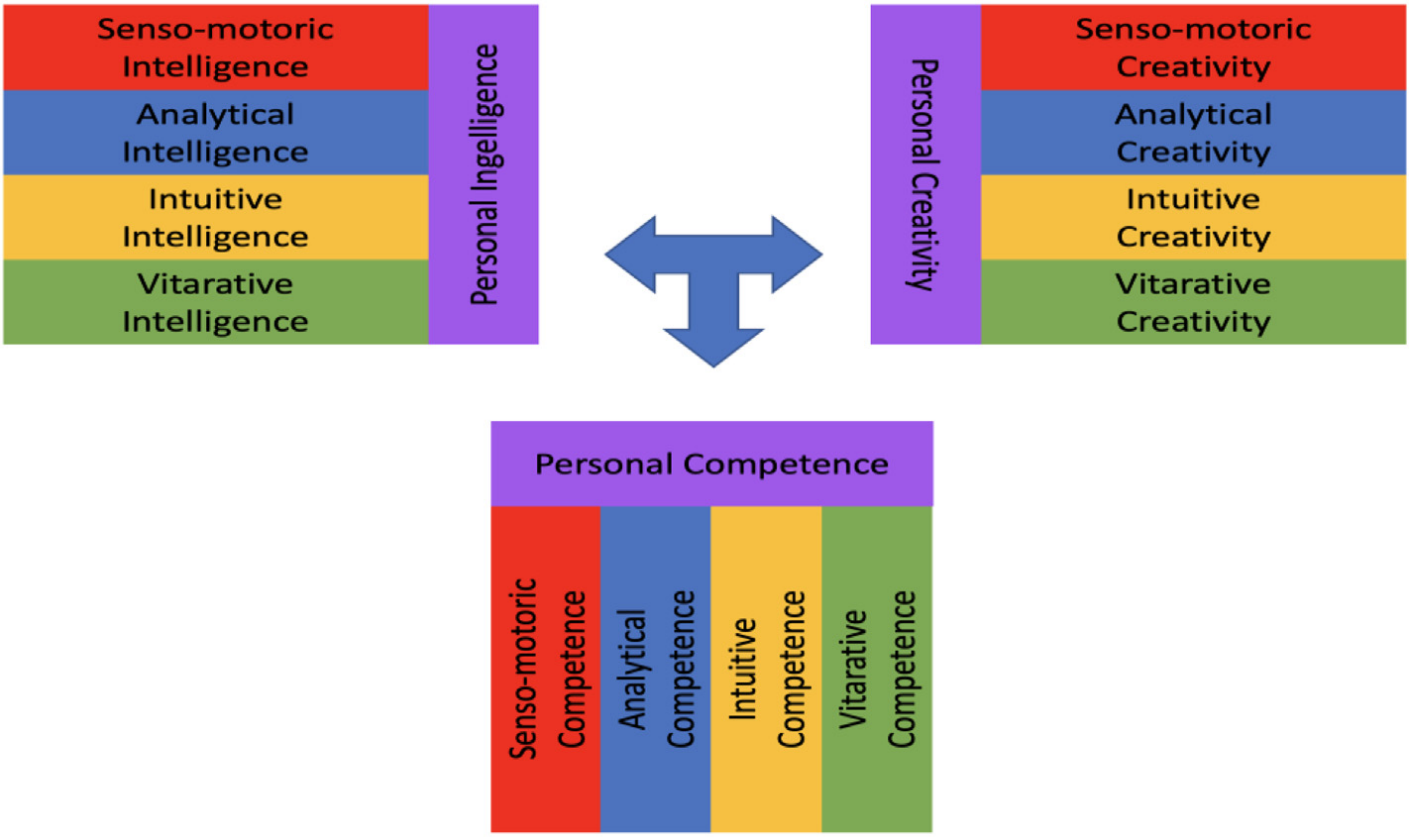
Interplay of intelligence, creativity and competence. Source: Own illustration adapted from (Müller and Rippel, 2011, p. 238).

With the help of intuition, inventors, for example, can anticipate new business fields. Such forward-looking actions cannot usually be planned purely rationally, but require an “intuitive feel”. Visions and imaginations open up options for action beyond the beaten solution paths. This is what we call “inspirational intuition”. This inspirational dimension of intuition has a long-term, holistic and gradual effect. Possible manifestations of inspirational intuition are inclusive dialog, increasing self-awareness, processing emotions, developing focus, refining the senses, and fostering ethicality (Jagtiani, 2018, p. 2, abstract). Mere rational thinking here would probably remain in old traditional patterns of action. The door for evolutionary or revolutionary ideas would remain closed without intuition. Fueled by intuition, creativity is the evolutionary force that drives adaptive change. It is a never-ending educational process that repeatedly produces innovative knowledge and action on the multidimensional basis of intelligence, creativity and competence (see Figure 5).

**Figure 6:**
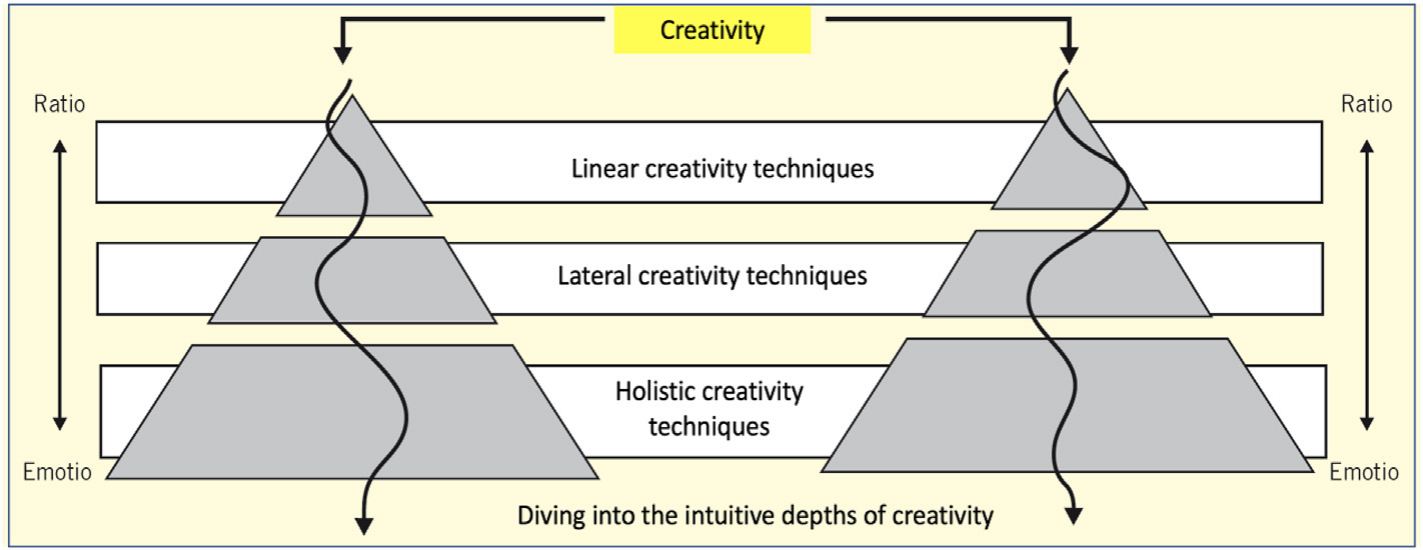
Step pyramid of creativity. Source: Own illustration adapted from (Müller and Rippel, 2011, p. 281).

Creativity techniques can be applied on the basis of one's own or collective creativity potential. In the simplest form, techniques are used that are based on linear thought structures. They generate linear creativity that draws on our existing knowledge. We move in a limited creativity space. Truly new things cannot emerge in this way. To expand the boundaries further, we can use creativity techniques that build on lateral thinking. New knowledge is generated primarily through associations and bisociations. A linking of concepts, images or ideas from different conceptual frames of reference takes place. Our horizon of observation becomes wider and the way is more open for new ideas. Holistic thinking structures can ultimately only be developed gradually; they require a long-term learning process. It requires nurturing and preparatory measures and techniques (see Figure 6). These include, for example, improved perception, a positive basic attitude, a strong mental focus, a high level of identification with the subject and a sense of what is possible. Our creativity thus spans a possible multi-level spectrum of creativity (Müller and Rippel, 2011, p. 240 ff.).

**Figure 7:**
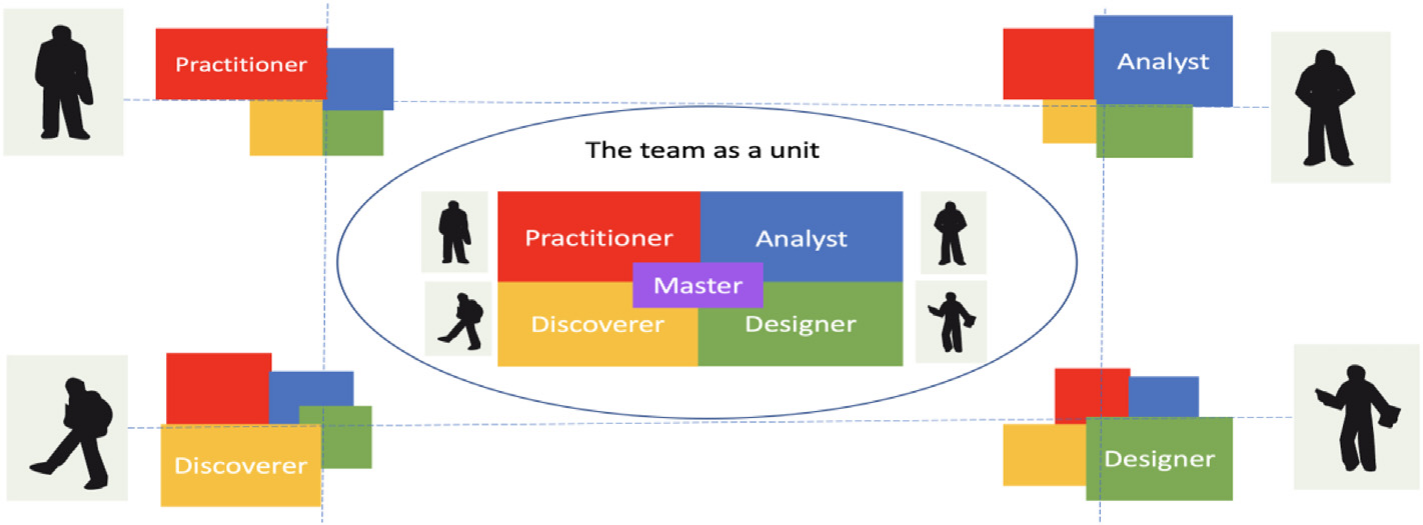
Matching of the inner and outer team. Source: Own illustration adapted from (Müller and Rippel, 2011, p. 293).

According to Gerd Binnig, the very own processes of isolation, attraction, reproduction, mutation and selection show themselves as processual foundations for creative processes (Binnig, 1989, p 143). Creative thought processes are also constantly reproduced, mutated and critically examined as thoughts and thought patterns in this general form. Based on our intelligences, creativities and competencies, different voices operate within us. The psychologist Friedemann Schulz von Thun coined the model of the “inner team” for dealing with our own human inner life. With the “inner team” model, he takes a closer look at the “inside” of communication. This is because we find togetherness and antagonism not only between people, but also within people. Most of the time we carry several souls in our chest (Schulz von Thun and Stegemann, 2008). According to the ideas of the CREA LEADERSHIP® competence model, different role models of the “inner team” work within us and speak with different “voices” (see Figure 7).

There is the “practitioner” with his sensory and motoric power. Our “analyst” who relies primarily on analytical thinking and shines through rationality and logic. The “discoverer” as the champion of creative intuition. He speaks from the imagination and conjures up initial visions and ideas. He relies primarily on inspirational intuition. The “designer” works for a harmonious whole and uses his emotional power of judgment. Still missing is the “master”, who finally listens to all individual actors and finally unites them. Comparable to a conductor, he brings together the individual internal “musicians” to form a coherent “orchestra”. The education of the individual voices is important for the maturing process of one's own personality, because each inner voice is connected with an individual benefit. In the educational process it is our task to recognize the strengths and their respective expressions, to develop them as needed and to use them in a goal-oriented manner. If one of the “inner voices” dominates us, a dominance profile is formed. This means that one can be more of a “practitioner”, the other more of a “discoverer”, “designer” or “analyst”. The dominance profile forms the starting point for one's own personality development. Knowing the different personality profiles also helps to perceive, reflect and appreciate the strengths and weaknesses of others. In this way, different talents can be synergistically combined to form an “outer team”. Müller and Rippel refer to this process as “matching.” (see Figure 7). The broader or deeper the individual's role models, the more diverse and valuable the differently perceived roles are for the “Outer Team.” The team member is versatile or specific. Through targeted role and competence management, the strengths and weaknesses of the individual team members and thus also of the entire team can be developed and controlled by the “master”. As a methodological approach to assessing competencies and creating a competency profile, reference is made here to the CREA LEADERSHIP® Competence Game according to Müller and Rippel (Müller and Rippel, 2014, p. 92–97). The competence game makes abilities and potentials visible. In this way, new possibilities can be recognized and creative strategies for competence development can be worked out. The game includes cards for the five basic competence dimensions (senso-motor, analytical, social, intuitive, personal) and 10 strengths and weaknesses for each dimension. With the help of the analysis, the main competence and a holistic overall profile should become apparent.

### 3.4 Invention and innovation

Innovation can be defined as “creating something new”. The root word comes from Latin and is made up of the two terms “novus” (new) and “innovatio” (something newly created). Schumpeter formulated a basic definition of innovation as early as 1911: „Innovation is the process of finding economic applications for technical inventions (Schumpeter, 1911, as cited in Scholtissek (2009), p. 163)”. Accordingly, every innovation is based on an idea or invention. However, the invention only becomes an innovation when it has been successfully marketed. An invention is therefore not an innovation per se. An invention only becomes an innovation when the inventor can assess the market value and benefits of his invention and has the drive to implement his idea in the market.

From the perspective of gaining knowledge, Schumpeter's original definition must be expanded to include the aspect of sustainability. At the time of Schumpeter, the topic of sustainability did not yet play a significant role. Our awareness and level of education have changed fundamentally. In the future, sustainable management will become an existential issue for mankind. From this perspective, mere market success must be critically questioned. In the future, not only “economic applications” will be required, but the ecological sense of the application must also be proven. The "economic applications“will become ”economical and sustainable applications. At the time, Schumpeter referred to technical invention.

From today's perspective, the spectrum of possible innovations has expanded significantly. In addition to the still important technical innovations (“technical inventions”), problem solving in general can be understood as an innovation approach today (“problem solving inventions”). The invention must therefore function as a problem solver. To be successful, the invention should have a uniqueness. Any uniqueness is therefore also pioneering and innovative. The unique innovation can no longer be related only to the product, but can be focused on other differentiating features. Today, for example, there are brand innovations such as Red Bull or distribution innovations such as Tupperware or Amazon in addition to pure technical inventions. In all innovations, it is clear that the innovation process basically consists of two subphases. On the one hand, there is the phase of invention (“problem solving inventions”) and, on the other, the phase of introduction and application (“economical and sustainable applications”).

**Figure 8:**
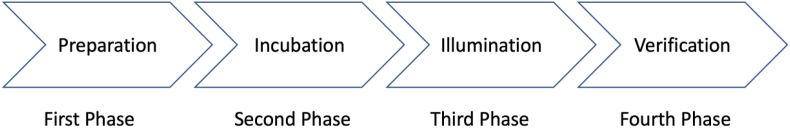
Systematic theory of creative thinking. Source: Own illustration based on (Wallas, 2015, p. 37).

In the groundbreaking theory of creative thinking according to Graham Wallas (see Figure 8), the acquisition of knowledge and the preparatory problem-solving phase also took up a lot of space. In the preparatory phase, the first step is to set the creative process in motion in order to initiate innovative knowledge. To this end, goals and expectations in particular are concretized, precisely formulated, timed and documented. This preliminary work forms the basis for generating ideas and concretizing innovations in the further course of the innovation process mainly by inspirational intuition.

Visionary and strategic actions by corporate leaders are always associated with dynamism and agility. On the one hand, visions and the strategic direction can be clearly planned and pursued; on the other hand, there must be openness and adaptability in order to be able to seize surprising opportunities and possibilities. Creative leaders become moderators between strategic planning and spontaneity. Recognizing the right moment - known as “kairos” in Greek - or the chance perception of opportunities (serendipity), sometimes determine success or failure, instead of foreseeable strategy. Socrates' saying “I know that I know nothing” is the prompt and the starting point for the search for new sources of knowledge and inspiring ideas. Based on the realization that existing knowledge is finite, it becomes necessary to constantly update and further develop existing knowledge. Knowledge can be mined and built up. However, knowledge can also decay and be lost. In addition to knowledge decay, knowledge can be completely destroyed and discarded. Many insights and skills are lost because they were only handed down in implicit form. In the context of knowledge management - as part of education - it is therefore necessary to identify knowledge, to salvage it, to make it explicitly accessible and thus transferable and learnable.

## 4 Discussion

Education, in the sense of shaping skills and abilities for the future, is an everlasting task. Both planned and disruptive changes require lifelong education and training. The increasing dynamics of the corporate environment and living conditions make an agile orientation necessary. Agility can be described as a form of strategy that makes it possible to anticipate challenges, shape the environment itself and thus create competitive advantages over other market players. Agility is therefore not just a form of flexibility, but the highest form of adaptability. The goal is to build a fast-learning organization that can adapt to changing circumstances at short notice and avoid organizational inertia (Sambamurthy et al., 2003, p. 238). Education is a key factor and access for innovative thinking and entrepreneurship. The importance of education, intuition, creativity and invention as precursors to innovation, as shown in the article, will continue to grow. People have to learn a new skill set increasingly accessible through digital technologies. And we need the time and funding to be able to pursue new opportunities. The World Economic Forum describes today's top five business skills as “active learning and learning strategies,” “analytical thinking and innovation,” “complex problem solving,” “critical thinking and analysis” and “creativity, originality and initiative” (World Economic Forum, 2020). For this reason, it is very important to recognize the role of education and inspirational intuition in these drivers of innovation and the great importance.

Intuition and creativity play an important role, as a creative catalyst, for the generation of innovations. In the interplay of perception, assessment and action, the necessary evolutionary further development takes place with the help of the human key competence “education”. Education and intuition thus become the creative path to innovation. There is a need for further research in the area of competence development. At German daycare centers, schools and universities, four types of competence in particular have been demanded and promoted so far: professional competence, methodological competence, social competence and personal competence. However, it is above all intuitive competence that should become the focus of scientific educational considerations. By expanding the previous competence models from four to five dimensions, including “intuitive competence”, a holistic approach to competence development is created (Müller and Rippel, 2011, p. 116). Previous competence profiles should include intuition and be made assessable and designable through self-assessment based on an Inspirational Intuition Inventory. A corresponding Inspirational Knowledge Imagination-Inventory (IKII) is currently under scientific development on the part of the author.

Intuition in the sense of an inspirational knowledge imagination can promote the development of new knowledge and build the intuitive bridge to an imaginary and divined world. An Inspirational Knowledge Imagination-Inventory (IKII) is to be developed to facilitate an assessment of the inspirational intuition of individuals. Based on a selective analysis of relevant literature, research and studies, a ten-question questionnaire was developed using a Likert scale to measure Inspirational Knowledge Imagination expression. The inventory is based on the assumption that people with high inspirational intuition are also more creative and innovative. In this way, such an inventory can help to improve the intuitive competence of organizations and their members. The Inspirational Knowledge Imagination-Inventory, for example, provides the starting point for in-depth psychological interviews and for the selection of intuitive and creative employees.”

It seems a central practical recommendation, using a combination of inspirational intuition and practical competency alignment and development, trainers are able to align and develop emerging leaders in a purposeful and effective way. In terms of a spiral curriculum according to Jerome Bruner, holistic competence development should begin in preschool and continue throughout life. With the help of spiral competence development, educational institutions and companies receive an instrument for competence-oriented education and training. By reflecting on aspired and existing competencies, a concrete and practice-oriented framework for action for the future is created. New requirements demand new knowledge and new skills. Innovative value-added contributions and competitive advantages emerge from visionary competencies. From inspirational intuition emerges an ongoing engine for dynamic competence development. The balance between sensing and intuition makes it possible to use the entire spectrum of rational and emotional perception and judgment for our holistic actions. Intuition and holistic creativity pave the way for us into a visionary world that does not yet exist. Albert Einstein called intuition a “sacred gift” and the rational mind a “faithful servant.” (Samples, 1976, p. 26). In the interplay of intuition and logic, new ideas and innovations open up again and again.

But what are the limits and possible dangers of this human creative power?

Yuval Noah Harari describes in his book “Homo Deus: A Brief History of Tomorrow.” the possibly imminent loss of control of the human species. Humans are not only changing their environment, but are also beginning to maximally change themselves, through artificial intelligence and biotechnology, thus creating a new world (Harari, 2015, p. 281 ff.) Derived from the biblical creation “And God blessed them, and said unto them, be fruitful, and multiply, and replenish the earth, and subdue it; and have dominion over the fish of the sea, and over the fowl of the air, and over every creeping thing that creepeth upon the earth.” (Genesis, chapter 1, verse 29; translated by the author mankind seems to have a divine mandate to lead. A mission to dominate, to protect and to preserve. The human race is now in danger of losing this divine leadership role in the future or giving it out of hand. We are reaching our humanistic limits and risk nothing less than the loss of human existence on earth. We accelerate the evolution more and more and strive for a change in the human being itself. An optimal development of the body, the brain and the knowledge. A super race that could be many times superior to today's humans. With abilities that were once considered divine. Humans could be preparing their own demise if these new “rulers of the earth” do not also undergo an evolution in tolerance, togetherness and harmony. There is no guarantee that there would be an existence protection for supposedly “inferior human life forms”. Unfortunately, the unrestricted affiliation with technology and the naive belief in the good in technological development could also be disappointed. Here, too, man's inner struggle between “good“ and “evil” is raging (Müller, 2021, p. 62 ff.) Thus, technological evolution can undoubtedly be used for the good of all mankind. The focus should be on supporting and promoting man, and man should always retain dominion over the "helpers" he has created. Otherwise, it could happen to him like the sorcerer's apprentice in the ballad by Johann Wolfgang von Goethe and the serving forces getting out of hand could easily lead to his downfall. Whether and to what extent this will happen certainly depends on how willing we humans are to give up our humanity and at the same time open ourselves up to a technological transhumanity. So far, technology has served us as a tool to support and improve life and as a chance for the future of humanity. If we cross the technological Rubicon to increasingly substitute and eliminate our humanity and human existence, the consequences for us will be unforeseeable. Ultimately, it would probably be tantamount to a renewed “expulsion from paradise”, because mankind again and irrevocably exceeds the limits of its own actions. Only this time, it would be the people themselves who banish themselves out of hubris, from the Garden of Eden for all time.

## 5 Conclusion

In summary, education and inspirational intuition are two central drivers of inventions and innovations. Education is understood as the transfer of explicit and implicit knowledge. Based on the fact that existing knowledge is finite and can become obsolete, it is necessary to keep developing knowledge. In addition to knowledge decay, knowledge can also be completely destroyed and discarded. Much knowledge is lost over time and escapes education because it has been passed down only in implicit form. It is therefore an important task to recover tacit knowledge and make it explicitly accessible.

The higher the level of innovation, the more important the new knowledge becomes compared to the previous knowledge. This is where the important role of inspirational intuition becomes apparent. Intuitive perception and emotive judgment are unlocking new ideas and generate new, unprecedented knowledge. Inspirational intuition and creativity thereby describe the ability to imagine what is possible. Knowledge transfer and knowledge exchange in the sense of education and knowledge exchange enable collective creativity for innovative impulses. With the help of intuition, new paths and new products and services can be sensed and created. Thus, education and inspirational intuition create a connection between the existing and accessible knowledge on the one hand and the possible knowledge of the future that can be intuited on the other hand.

## Declarations

### Author contribution statement

Jochem Müller developed and the wrote this article.

### Funding statement

This research did not receive any specific grant from funding agencies in the public, commercial, or not-for-profit sectors.

### Data availability statement

No data was used for the research described in the article.

### Declaration of interests statement

The authors declare no conflict of interest.

### Additional information

No additional information is available for this paper.

## Footnotes

1 Gardner differentiates among others into linguistic intelligence, logical-mathematical intelligence, visual-spatial intelligence, musical intelligence, physical-kinaesthetic intelligence, social-interpersonal intelligence, social-intrapersonal intelligence, naturalistic intelligence, synthesizing intelligence, creative intelligence, respectful intelligence, ethical intelligence (Gardner 1983, p. 60 ff.).
